# From sample to sonde to Sentinel-2: insights from a multi-scale chlorophyll-*a* monitoring effort in the Hudson River, New York

**DOI:** 10.1007/s10661-025-14844-3

**Published:** 2025-12-09

**Authors:** Wilson B. Salls, Robert J. Welk, Tyler V. King, Natasha A. Scavotto, Rebecca M. Gorney, Sabina R. Gifford, Michael D. Stouder, Elizabeth A. Nystrom, Jennifer L. Graham

**Affiliations:** 1 New York Water Science Center, U.S. Geological Survey, Troy, NY USA; 2 Water Resources Mission Area, U.S. Geological Survey, Boise, ID USA

**Keywords:** Chlorophyll-*a*, Remote sensing, Rivers, Fluorescence, Random forest modeling, Machine learning

## Abstract

Monitoring cyanobacteria and other nuisance phytoplankton in the Hudson River is of great interest given its societal and ecological importance. Satellite remote sensing provides a cost-effective method to monitor chlorophyll-*a* (chl-a), a common proxy for algal biomass; however, the dynamic nature of rivers complicates approaches traditionally applied to lakes and oceans. During 2021–2023, we collected discrete samples for laboratory measurement of chl-a and measured in situ chl-a fluorescence during a series of longitudinal boat surveys along a 220-km reach of the lower Hudson River. Surveys were timed to coincide with Sentinel-2 satellite overpasses. We first investigated relations between laboratory-measured chl-a concentration and field-measured chl-a fluorescence, observing a weak correlation (*r*^2^ = 0.25) that improved substantially after splitting data by day (mean *r*^2^ = 0.53). Separately, to estimate chl-a fluorescence using satellite data, we developed a series of random forest models leveraging the rich fluorescence dataset collected. We tested three model types: individual day models, leave-one-out models trained on all days except a holdout test day, and a single pooled model trained on all days. Generally, individual day models exhibited lowest error (mean of mean absolute error [MAE] = 0.16 relative fluorescence units [RFU]), followed by the single pooled model (MAE = 0.22 RFU). Daily holdout models showed highest error (mean MAE = 0.40 RFU); this approach was intended to represent model performance on a day unseen in the training set, providing a more conservative estimate of performance than the more traditional pooled approach. Findings from both analyses emphasize the importance of considering temporal variability when modeling riverine systems.

## Introduction

Large rivers are important for drinking water, recreation, and navigation, and serve as critical habitat for a diverse array of flora and fauna. Excessive algal growth, cyanobacteria, and cyanotoxins occur in many large United States rivers (Graham et al., 2020; Linz et al., 2023). Cyanotoxins can pose threats to human and animal health, particularly in rivers used as public water supplies and for primary contact recreation such as swimming. Algal bloom indicator thresholds that are used to take action for public health protection are not applied consistently across the U.S., posing challenges—especially in riverine systems, which often cross political boundaries (Nietch et al., 2022; Stackpoole et al., 2024). Some jurisdictions rely on cyanotoxin, chlorophyll*-a* (chl-a), or cyanobacterial concentration while others determine health risks based on visually apparent algal accumulation such as surface scums or green discoloration (Chaffin et al., 2021; Clark et al., 2017; Gorney et al., 2023). Chl-a can be effectively used as a proxy for algal biomass (Peipoch & Ensign, 2022) and for harmful algal blooms in freshwaters when algal assemblages are dominated by cyanobacteria (Stumpf et al., 2016).

Chl-a data are typically obtained either by collection of discrete samples that are then analyzed in the laboratory, here termed laboratory-measured chl-a, or with sensor-based measurement of fluorescence, here termed field-measured fluorescence (Foster et al., 2022). There are several ways to collect data with each of these methods; for example, point sampling that represents one depth and location, integrated depth sampling that represents a vertical composite at one location, and continuous transect sampling—often performed via boat surveys—that represents surface measurements along the survey path (Crawford et al., 2015; Foster et al., 2022). These sampling approaches can be combined to meet research and monitoring objectives but run the risk of missing important water quality signals because they are limited in space and time. Additionally, different measurement methods that occur at the same time and place may yield different results, complicating interpretation of monitoring efforts (Gregor & Maršálek, 2004; Trees et al., 1985). Laboratory-measured chl-a and field-measured chl-a fluorescence are two fundamentally different parameters and are often, but not always, strongly correlated (Foster et al., 2022). While laboratory-measured chl-a is generally considered to provide accurate, consistent results, field-measured fluorescence can be confounded by various factors, including variations in algal community composition (Prestigiacomo et al., 2022; Proctor & Roesler, 2010), algal health (Olaizola et al., 1994), optical interferences from materials present in the water column known as matrix effects (Pellerin et al., 2013), and non-photochemical quenching (Beutler et al., 2002). For these reasons, derivation of chl-a concentration by converting from field-measured fluorometric response tends to be biased and unreliable (Liu & Georgakakos, 2021). However, fluorescence measurements can be collected readily at low cost relative to laboratory measurements, enabling construction of datasets dense in time and/or space (Gall & Davies-Colley, 2021).

Satellite remote sensing has emerged as a means to complement field-based water quality monitoring. Though satellite measurements are also prone to error, they offer a method of expanding data coverage in both space and time that is often more cost-efficient for users. Several satellite platforms can detect chl-a, each with its own set of spatial, temporal, and spectral resolutions. One platform commonly used for monitoring phytoplankton, particularly cyanobacteria, is the European Space Agency (ESA) Ocean and Land Colour Instrument (OLCI) onboard the Sentinel-3 satellite mission (Konik et al., 2023; Wang et al., 2023; Wynne et al., 2021). With daily coverage and the ability to spectrally differentiate cyanobacteria from other phytoplankton, OLCI’s value is underscored by its adoption in programs such as the Cyanobacteria Assessment Network (CyAN), which produces a cyanobacteria data product widely utilized by federal and state agencies (Schaeffer et al., 2018). However, OLCI’s pixel resolution of 300 m limits its application to only larger aquatic systems, with relatively poor coverage in rivers. The complementary MultiSpectral Instrument (MSI) onboard the Sentinel-2 satellite boasts substantially smaller pixel size (10, 20, or 60 m depending on waveband), with the tradeoff of a longer 5-day repeat period. Sentinel-2 possesses fewer spectral bands and is thus unable to distinguish cyanobacteria from other phytoplankton, but has been demonstrated for estimation of chl-a (Llodrà-Llabrés et al., 2023) in most U.S. lakes and many rivers that are too small to be resolved by OLCI (Clark et al., 2017).

Several methods have been developed for retrieving chl-a from multispectral satellite imagery (Yang et al., 2022). Most algorithms yield a spectral index, which then must be related to the parameter of interest—including chl-a—using an empirical model, most commonly a regression model. The Maximum Chlorophyll Index (MCI) (Gower et al., 2005) and Normalized Difference Chlorophyll Index (NDCI) (Mishra & Mishra, 2012) are two common spectral indices, and various studies have calculated linear regression coefficients to relate these indices to chl-a (Akbarnejad Nesheli et al., 2024; Ansper & Alikas, 2019; Johansen et al., 2024; Salls et al., 2024). Another method is to input spectral data directly into a model—in many cases, a machine learning model such as a neural network or random forest—fitting field-collected chl-a concentrations to the suite of wavebands as predictor variables (Khan et al., 2022; Pahlevan et al., 2020; Saberioon et al., 2020).

A major consideration when constructing any satellite retrieval model is selection of training data. The ideal approach would be to train a new model for each satellite overpass to account for variation in atmospheric and matrix effects; however, collecting such frequent field data tends to be cost prohibitive and minimizes the benefit of high observation frequency offered by satellite remote sensing. For this reason, a common approach is to build a single, pooled model using data from all available days—often reserving a portion for validation—to apply to future satellite observations moving forward (Johansen et al., 2024; Salls et al., 2024; Seegers et al., 2021). The pooled model approach is subject to temporal variations in algal community composition, water quality, and atmospheric parameters that may interfere with chl-a retrieval (Niroumand-Jadidi & Bovolo, 2022; Pahlevan et al., 2021; Pan et al., 2022), potentially introducing greater error than would exist in a model built for an individual day. The highly dynamic settings of rivers are particularly sensitive to this type of error (Xiao et al., 2023). Thus, there is a tradeoff between minimizing cost of data collection and maximizing model predictive power.

Another important decision when modeling water quality with satellite imagery is selection of a satellite product and processing level. MSI image processing levels include top of atmosphere (TOA, also called Level 1C) with no atmospheric correction applied and two atmospheric correction methods (both considered Level 2A): surface reflectance (SR) produced with terrestrially focused Dark Dense Vegetation (DDV) atmospheric correction (Richter et al., 2012) and aquatic reflectance (AR) focused dark spectrum fitting atmospheric correction (Vanhellemont & Ruddick, 2018). Each approach has tradeoffs between availability and accuracy, with the TOA and SR produced operationally by the European Space Agency, and AR produced operationally for the conterminous United States by the U.S. Geological Survey (King et al., 2024).

The three satellite products differ in terms of whether and how they correct for atmospheric constituents—aerosols and gases—that interfere with the signal from the target—in this case, light emitted from the water’s surface. Estimating aerosol content is generally done by identifying “dark targets” in TOA imagery and iterating through a set of aerosol models to minimize the difference between observed and computed radiance values. Sen2Cor is perhaps the most widely used atmospheric correction for Sentinel-2 imagery because it is applied by the ESA to produce the Level 2A bottom of atmosphere surface reflectance (Richter et al., 2012). Sen2Cor uses a DDV approach adapted from Kaufman and Sendra (1988) where the pixels with the lowest at-sensor radiance in the shortwave infrared (SWIR; MSI Band 12) are identified as the dark targets. This approach was developed for terrestrial applications and is therefore referred to as surface reflectance (SR). In contrast, the Atmospheric Correction for Operational Land Imager (OLI) Lite (ACOLITE) processor identifies pixels with minimum radiance for all bands, allowing different darkest pixels to be selected for each band (Vanhellemont & Ruddick, 2018). This can become important when turbid water is identified as the darkest SWIR pixels in the DDV approach implemented in Sen2Cor because turbid water may have the lowest SWIR reflectance but not the lowest reflectance for visible light. ACOLITE was developed specifically to address challenges in retrieving reflectance values over water with the DDV approach, and the output is therefore referred to as AR. Though AR and SR are both intended to correct for the interfering signals of atmospheric constituents, the process of atmospheric correction can introduce large errors (Gordon & Wang, 1994), leading some users to opt for raw TOA imagery. At the time of this study, there is not a clear consensus on whether AR, SR, or TOA yields superior performance, and performance across various atmospheric correction methods is highly dependent on environmental factors (Grendaitė & Stonevičius, 2022; Pahlevan et al., 2021). Therefore, many factors must be considered when selecting a satellite product to address specific research objectives.

Remote sensing of rivers presents unique challenges compared to that of marine and lake environments. Water quality in rivers can be highly temporally dynamic given the lotic nature of these systems and is subject to rapid changes with hydrologic conditions (Tan et al., 2016). Additionally, rivers tend to be narrower than other systems, presenting a greater risk of adjacency effects from land (Zhao et al., 2024). Recent advances in adjacency correction algorithms have been made (Castagna & Vanhellemont, 2025; Pan & Bélanger, 2025; Wu et al., 2024), but further work is needed to address the unique challenges and develop remote sensing applications for rivers.

Past work has successfully applied various remote sensing methods to augment chl-a monitoring efforts. However, there remains a need to better understand how various methodological choices influence overall model efficacy, and how sampling efforts can be optimized. Here, we build on existing work (King et al., 2022) to integrate information from multiple approaches in deriving information about chl-a using machine learning methods. We demonstrate a method for developing a site-specific relation between satellite data and in situ field-measured chl-a fluorescence, a parameter that is easily measured and widely available. In developing this relation, we investigate the impacts of various factors on model success.

**Fig. 1:**
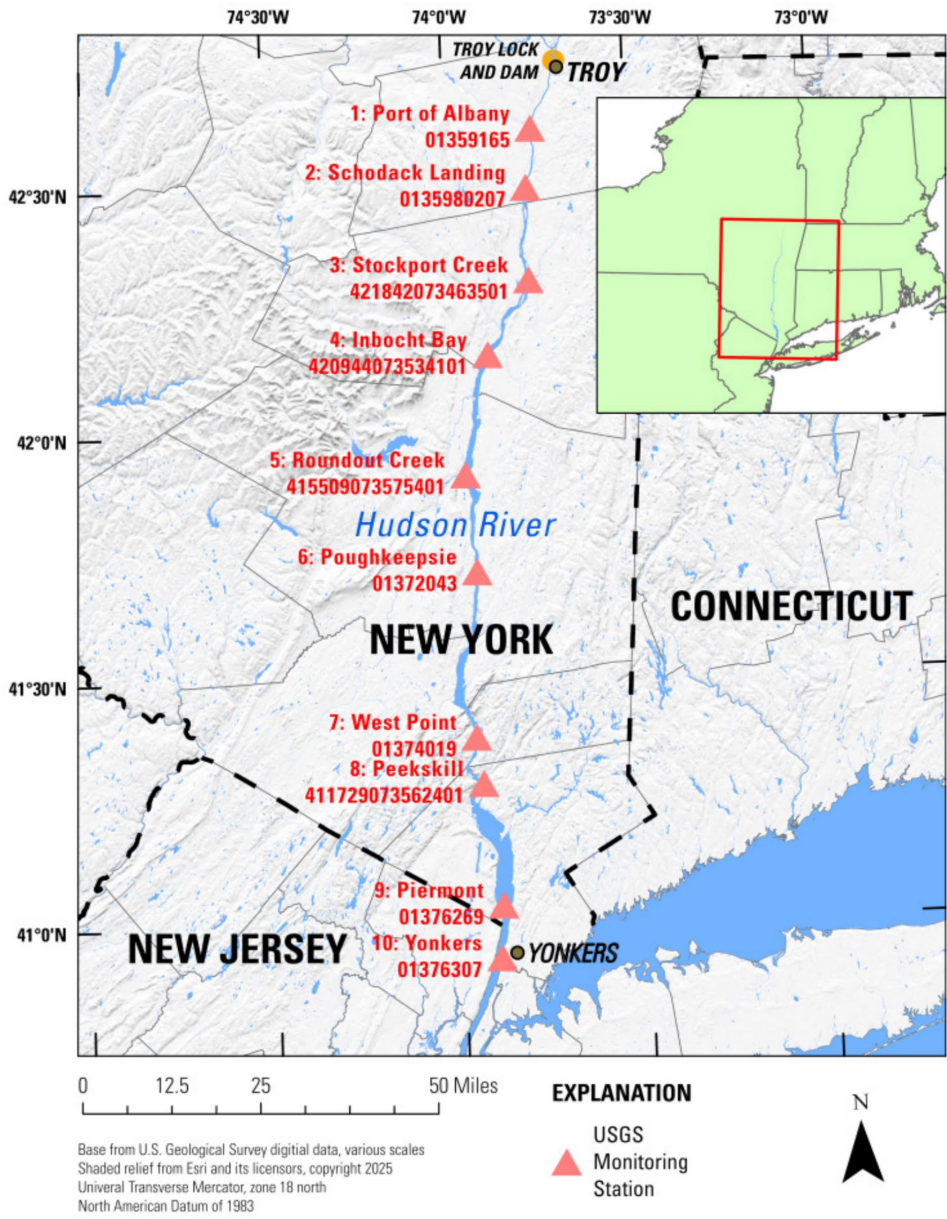
Map of the study area showing the 10 U.S. Geological Survey (USGS) sites (U.S. Geological Survey, 2024) at which discrete water samples were collected for laboratory measurements of chlorophyll*-a*. Inset map shows location within the northeastern U.S. Longitudinal boat surveys were conducted between Troy and Yonkers

We chose the lower Hudson River as a study location given its societal, economic, and ecological importance to the northeastern U.S. The lower Hudson River (from Troy to the City of New York, NY; Fig. 1) has a rich historical data archive and ongoing data collection, including at several near-shore point locations that are continuously monitoring chl-a and other parameters through the Hudson River Environmental Conditions Observing System (HRECOS) program (*Hudson River Environmental Conditions Observing System*, 2023). Such efforts provide temporally dense water quality data at relatively few discrete points in space. The ability to spatially interpolate between monitoring sites across the entire lower Hudson River would provide better understanding of spatiotemporal variability and environmental conditions driving water-quality dynamics.

In this study, we establish a relation between satellite observations and field-measured data to build a chl-a model for the lower Hudson River. We then go on to explore (1) the degree to which field-measured fluorescence data reflect actual variations in laboratory-measured chl-a concentration; (2) differences in performance between models trained for individual days, models trained on all days (with one survey held out for evaluation, here termed “testing”), and a pooled model trained with data from all days; and (3) the influence of atmospheric correction on model performance and generalizability. In addition to yielding an application-ready chl-a model for the lower Hudson River, our results provide important considerations for future studies seeking to integrate remote sensing into chl-a monitoring programs.

## Materials and methods

### Study site

This study was conducted along 220 km of the lower Hudson River, specifically, from Troy, NY, to Yonkers, NY (Fig. 1). As described below, both continuous field measurements and a series of water samples were collected along this part of the river. The net river flow is southward and is tidally influenced until a dam in Troy located just upriver of Site 1 (Fig. 1).

### Field data

Two types of water quality data were collected concurrently along the lower Hudson River during boat surveys: (1) continuous field-measured longitudinal data and (2) discrete surface samples used to generate laboratory-measured data from 10 discrete sites (Fig. 1). Data collection occurred during 12 surveys conducted in the summer and fall of 2021, 2022, and 2023. Not all sites were sampled on all dates—site counts by date are shown in Results. Field-measured data are available in Welk et al. (2025), and laboratory-measured data are available from the Water Data for the Nation platform (U.S. Geological Survey, 2024).

Continuous longitudinal field measurements were collected every 15 s using an EXO2 (YSI, Inc., Yellow Springs, OH) sonde attached to a flow-through cell with through-hull water access drawing from a depth of approximately 0.5 m below the water surface. Water was pushed through the flow-through cell by the movement of the boat; in no wake zones, a pump was used. The sonde recorded several water quality parameters (water temperature, specific conductance, dissolved oxygen, turbidity, fluorescent dissolved organic matter [fDOM], and chl-a fluorescence in relative fluorescence units [RFU] using the YSI Total Algae Sensor). Only chl-a fluorescence is discussed herein; all data are available in Welk et al. (2025). Though different individual sensors were used over the course of the 3-year study period, each was calibrated and operated in accordance with USGS protocols (Foster et al., 2022; Wagner et al., 2006) prior to each field date to ensure consistent and accurate measurement across all collection campaigns. GPS data were collected using a Garmin International, Inc. (Olathe, KS) GPSMAP 942xs (2021) or Garmin GPSMAP 64 s (2022–2023) and were related to continuous data using time stamps. Time stamps were synchronized between the sonde and GPS handheld units before each sampling trip.

Discrete surface samples were collected for chl-a analysis at 10 sites (Fig. 1). As the boat approached each sampling location, water passing through the flow-through cell was briefly partially diverted from the chamber to fill sample bottles. Samples were field filtered when conditions allowed or filtered upon return to the laboratory. The volume of water filtered depended on the amount of suspended material in the sample, generally 100–250 mL, using 47-mm glass fiber filters, which were then shipped frozen on dry ice to the USGS National Water Quality Laboratory (Denver, CO). The filters were analyzed fluorometrically (excitation value of approximately 430 nm) using the acidified U.S. Environmental Protection Agency (EPA) Method 445.0 (Arar & Collins, 1997) with a minimum reporting level of 0.10 µg L^−1^.

### Remote sensing data

Level 1 C (TOA) and Level 2A (SR) imagery collected with the MSI sensors on the Sentinel-2A and Sentinel-2B satellites for tiles 18TWN, 18TWM, and 18TWL were obtained from the ESA through the Copernicus Application Programming Interface (ESA, 2022) for the 12 sampling dates. Imagery on one date (8/25/22) was completely obscured by clouds and was excluded from further analysis. Each image was masked to include only pixels within the high-water bounds of the Hudson River, as defined by the National Hydrography Dataset Plus version 2 (NHDPlusV2) (McKay et al.,^2012^) that were mapped as the open water category (Class 6) by the Sentinel-2 scene classification layer provided by ESA. The three tiles were merged prior to atmospheric correction.

There are multiple atmospheric correction approaches for earth observing satellite imagery. Here, we test two—the SR approach provided as the Level 2A product from ESA, and the AR Dark Spectrum Fitting Approach implemented in Atmospheric Correction for OLI “lite” (ACOLITE) generic processor version (v 20,221,114.0) (Vanhellemont, 2019). Refer to Vanhellemont and Ruddick (2018) for a full description of the dark spectrum fitting approach. The default ACOLITE settings were used in the atmospheric correction.

### Data analysis

**Fig. 2:**
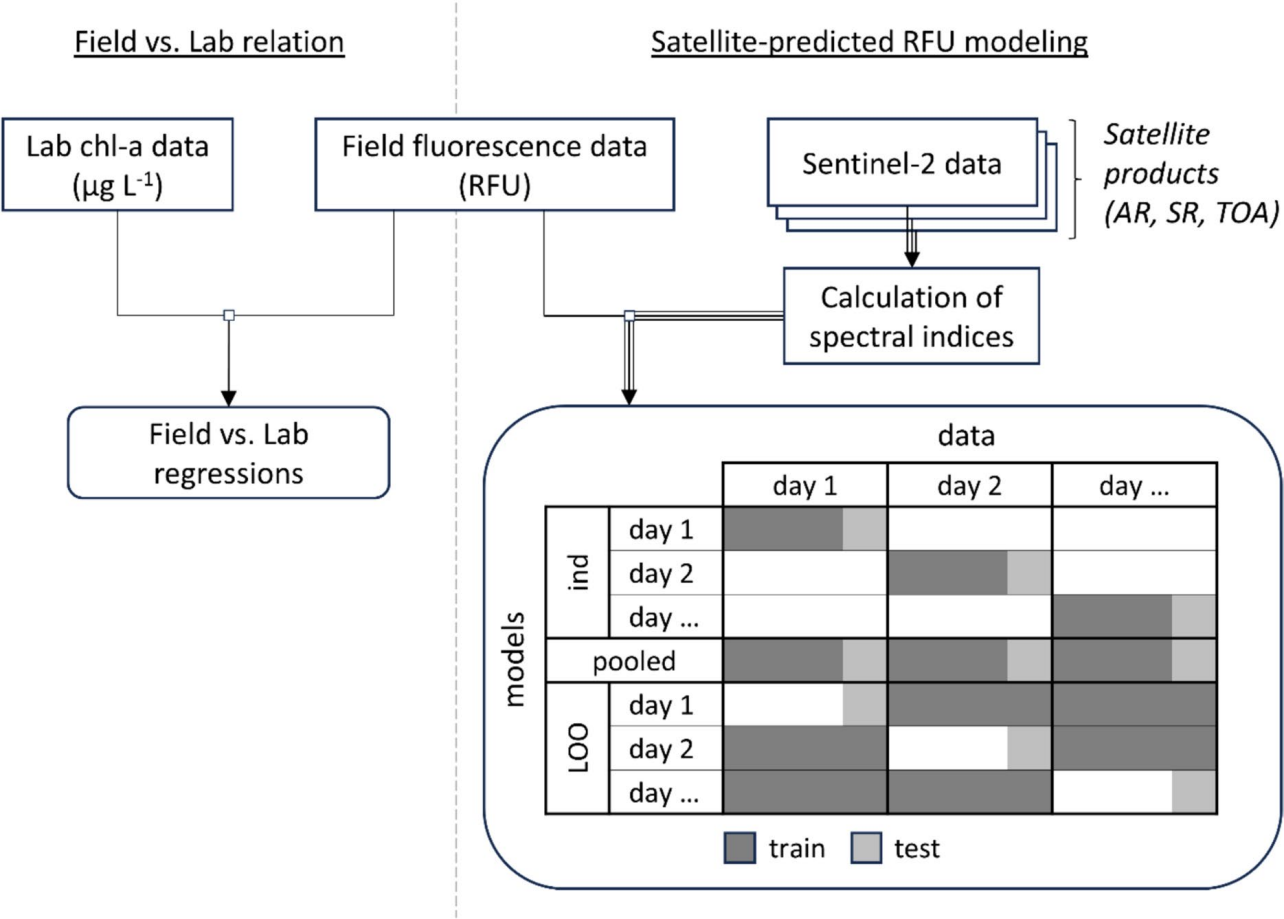
Schematic diagram showing data and modeling analyses. We matched laboratory- and field-measured data to investigate the relation between the two. Separately, we investigated three types of models to relate Sentinel-2 reflectance data to field-measured chlorophyll-*a* (chl-a) fluorescence data: individual day models (ind) (one for each day), leave-one-out (LOO) models (one for each day), and a pooled model for all days. These same models were investigated across aquatic reflectance (AR), surface reflectance (SR), and top of atmosphere (TOA) products

Broadly speaking, the study comprised two analyses: (1) relating field-measured chl-a fluorescence in RFU to laboratory-measured chl-a in µg L^−1^ and, separately, (2) building and evaluating a series of models to estimate chl-a fluorescence in RFU based on Sentinel-2 imagery (Fig. 2). Note that the two analyses were completely separate and not used in any way to inform each other; the initial analysis was done simply to investigate strength or weakness of the relation between field-measured fluorescence and laboratory-measured chl-a concentration in our study system, not to inform satellite models.

To assess the degree to which variations in field-measured RFU values represented laboratory-measured chl-a values, the two types of samples were matched spatially based on GPS location. A 0.25-mile (0.4-km) radius around each site location was established, and all field measurements taken within that buffer were aggregated using the median RFU value to match with the single concurrent laboratory-measured chl-a value. We applied this buffer to account for uncertainty stemming from latency between the time the water entered the intake and the time it passed over the sensor after flowing through connective tubing; we selected a spatial buffer as opposed to a temporal one to account for varying boat speeds, standardizing the boat track distance as opposed to an amount of time associated with an individual site. We then established linear regressions to relate RFU to chl-a—one with all data, one for each site, and one for each day. We assessed correlation strength using adjusted *r*^2^ values.

To prepare for model construction, Sentinel-2 images collected within three calendar days before or after field campaigns were paired with field-measured chl-a fluorescence data. Single pixel values were extracted and matched with field-measured chl-a fluorescence observations for each of the MSI bands. Single pixel extraction was selected as opposed to pixel arrays because, given the spatial density of field measurements, arrays from adjacent field measurements would have an increased risk of containing overlapping sets of pixels that introduce spatial autocorrelation issues. Several previously demonstrated spectral indices (Table 1) were computed from the reflectance values and matched with chl-a fluorescence values to produce a paired dataset to train and test remote sensing models (Welk et al., 2025). Note that models were not informed by the separate part of the study investigating the relation between field-measured fluorescence and laboratory-measured chl-a mentioned above.

**Table 1:** Spectral indices and equations used for random forest regression

	Index name	Equation	Citation
S01	Be162sub_Beck_2017	$b5-b4$	Beck et al. (2017)
S02	BR23_OReilly_1998	$\frac{b2}{b3}$	O’Reilly et al. (1998)
S03	BR54_Gons_2002	$\frac{b5}{b4}$	Gons et al. (2002)
S04	BR8a4_Tebbs_2013	$\frac{b8a}{b4}$	(Tebbs et al., 2013)
S05	Go04MCI_Gower_2004	$b5-b6$	Gower et al. (2005)
S06	KIVU_Beck_2016	$\frac{b2-b4}{b3}$	Beck et al. (2016)
S07	L83BDA_Beck_2016	$\left(\frac{1}{b2}-\frac{1}{b4} \right)*b3$	Beck et al. (2016)
S09	MCI_Le_2013	$b5-b4-\left(b6-b4\right)*\left( \frac{704.1-664.6}{740.5-664.6}\right)$	Le et al. (2013)
S10	Moses3b_Moses_2012	$\left(\frac{1}{b4}-\frac{1}{b5}\right)*b6$	Moses et al. (2012)
S11	NDCI_Mishra_2012	$\frac{b5-b4}{b5-b4}$	Mishra and Mishra (2012)
S12	NDCI_Beck_2016	$\frac{b8a-b4}{b8a+b4}$	Beck et al. (2016)
S13	S23BDA_Beck_2016	$\left(\frac{1}{b4}-\frac{1}{b5} \right)*8$	Beck et al. (2016)
S14	S2FLHBlue_Beck_2016	$b3-\left(b4+\left(b2-b4\right)\right)$	Beck et al. (2016)
S15	SABI_Beck_2016	$\frac{b8a-b4}{b2+b3}$	Beck et al. (2016)
S16	Toming_2016	$b5-\left(\frac{b4+b6}{2}\right)$	Toming et al. (2016)
S17	ZhFLH_Zhao_2010	$b8a-\left(b5+\left(b4-b5\right)\right)$	Zhao et al. (2010)

#### Modeling

We trained and tested a series of random forest models, each predicting chl-a fluorescence (given in RFU) as the response variable and using the suite of all 17 satellite spectral indices as predictor variables. Three types of models were trained: (1) *individual models* trained for each of the 11 surveys within 3 days of satellite imagery (11 models); (2) *leave-one-out *(LOO) models, wherein each model was trained on data from all but one of the surveys, which was left out as testing data (11 models); and (3) a *pooled model* trained and tested using data from all days together—totaling 23 model configurations. The individual models were trained to evaluate ability to interpolate water quality conditions for a single date, the LOO models were trained to approximate the accuracy of extrapolating models to conditions outside those in the training data, and the pooled model was used to evaluate a common approach taken in validating satellite monitoring efforts.

For each survey date, we split observations into training and test sets using a 75%/25% split stratified by field-measured chl-a fluorescence values to ensure common distributions of the target variable in each set. These splits were applied to each individual day model, as well as the pooled model for all days. LOO models were trained on all data from all days besides the test day and tested on only the same 25% of data from the test day used to test the individual day and pooled models (refer to Fig. 2). The same 25% of data from each day was used to test all three model types to remove variability resulting from using inconsistent testing data, ensuring comparability between model metrics across the three model types. Finally, the 23 model configurations were applied to each of the three remote sensing products (AR, SR, TOA), resulting in a total of 69 models.

Cross-validation was used to tune three random forest hyperparameters—number of trees, tree depth, and number of variables. Observations were spatially clustered into one of eight cross-validation folds based on location to reduce the influence of spatial autocorrelation. Using the set of optimized hyperparameters, models were fit to each of the training datasets. Test set predictions were evaluated by use of empirical distribution matching (EDM)—a bias correction approach commonly used to improve accuracy in ensemble tree models (Belitz & Stackelberg, 2021). This method provides test set predictions with an empirical cumulative distribution function (ECDF) identical to the ECDF of the observed data.

Satellite models were assessed using mean absolute error (MAE) and bias metrics, which, for this purpose, are preferred over coefficient of determination (*r*^2^) from linear regression of modeled and observed values because they do not require the assumptions of normality and homoskedasticity and are comparable across different data ranges (Seegers et al., 2021). To compare model types and satellite products, we calculated mean values across days for these metrics; in those cases, we used absolute value of bias instead of raw bias to retain bias magnitude, regardless of whether negative or positive. Additionally, the three satellite product types were ranked within each of the 23 sets of models, and mean ranks for each product were calculated to evaluate differences in performance. Mean rank was calculated by assigning ranks to each satellite product for each day—where 1 was given to the product with the lowest MAE and 3 to the one with the highest—and then averaging these ranks across all days; thus, a lower mean rank value indicates better performance.

### Mapping

To demonstrate application of the models developed here, we applied models to each MSI image to generate estimates of chl-a fluorescence. Spectral indices were input into the individual day model for each day to yield modeled RFU values across all river pixels on that date. Modeled chl-a fluorescence rasters were produced by applying the selected random forest model to the spectral index predictor variable across all non-cloudy river pixels. These rasters were mapped and investigated for spatial and temporal patterns.

## Results

Across all dates, field-measured chl-a fluorescence observations that were matched with satellite observations ranged from 0 to 4.6 RFU (mean: 0.85 RFU, median: 0.78 RFU). Field-measured chl-a fluorescence observations that were matched with laboratory-measured samples ranged from 0.03 to 4.91 RFU (mean: 0.81 RFU, median: 0.65 RFU). Laboratory-measured samples that were matched with field-measured chl-a fluorescence observations ranged from 0.26 to 22.34 µg L^−1^ (mean: 3.64 µg L^−1^, median: 2.72 µg L^−1^).

### Comparison of field and laboratory measurements

**Fig. 3:**
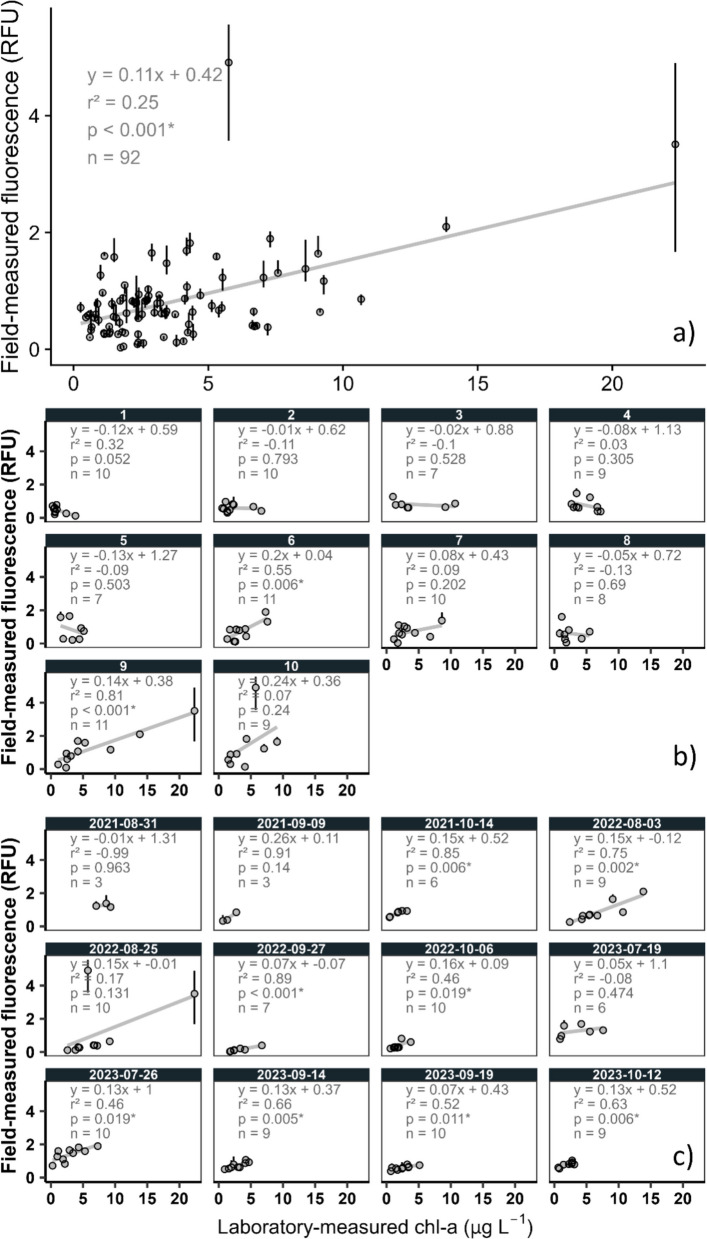
Regression models showing relations between chlorophyll-*a* (chl-a) fluorescence collected by field-measured (in relative fluorescence units [RFU]) and laboratory-measured (lab) chl-a (in µg L^−^.^1^): **a** for all survey dates and sites, **b** by survey site (refer to Fig. 1 for corresponding site names), and **c** by date. Vertical bars around points indicate range of field-measured fluorescence values associated with a given sample collection. * denotes a statistically significant relation at *p* < 0.05

To understand the relation between field- and laboratory-measured chl-a, we used regression models to relate median field-measured RFU values to chl-a concentrations (µg L^−1^). In a pooled regression model that included all sites and dates, only a small part of variance was explained (*r*^2^ = 0.25), but the relation was positive (slope = 0.11) and statistically significant (*p* < 0.001; Fig. 3a). For individual site models, correlations were also relatively weak (Fig. 3b). The mean *r*^2^ value was 0.14 (mean *r*^2^ = 0.19 with negative *r*^2^ values set to 0). Only 2 of the 10 site-specific models were statistically significant (*p* < 0.05). Slopes were variable among models, ranging from − 0.12 to 0.24 and including six negative values. For date-specific models, relations were stronger (Fig. 3c), with eight of the 12 dates showing statistical significance at *p* < 0.05. These models tended to explain more variance (mean *r*^2^ = 0.44; mean *r*^2^ = 0.53 with negative *r*^2^ values set to 0) than site-specific models. All slopes were positive, ranging from 0.05 to 0.26, except for one model that had limited data and a small range of values.

The dataset included an apparent outlier with a high chl-a fluorescence value (4.73 RFU); we chose to retain this observation in the final analysis because we could not identify any functional reason to remove it; however, this data point substantially influenced model results. When this data point was excluded from analysis, the pooled regression model explained more variance (*r*^2^ = 0.35; *p* < 0.001). The model for Site 10 also explained more variance but still was not statistically significant (*r*^2^ = 0.28; *p* = 0.1), while the model for 8/25/2022 explained more variance and was statistically significant (*r*^2^ = 0.95; *p* < 0.001).

### Modeling

The number of observations in training and testing sets for each day ranged from 142 to 1058 and 50 to 356, respectively (Table 2). For the LOO model on a given day, observation count for the training set is the sum of training and test sets from all days besides that one; likewise, observation count for the test set is the same as sum of training and test set counts from the individual model for that day. For the pooled model for all days, observation counts are the sums of observations across all days for training and testing sets, respectively. The distributions of chl-a fluorescence values in the training and testing sets were similar within each individual day and within the pooled model for all days, as were modeled results for AR, SR, and TOA (Appendix 1: Fig. 8).

**Table 2:** Summary of field survey data collected in the lower Hudson River, including mean, standard deviation (SD), and observation count (*n*) for each date and across all days, along with timing of corresponding satellite imagery. Data used to train satellite models and data used to compare field-measured fluorescence and laboratory-measured chl-a are summarized separately. Note that 8/25/22 was only used in the comparison of field and laboratory measurements, not in modeling, due to cloud cover in the satellite image

Survey date	Image date	Latency (days)	Field-satellite matches				Field-laboratory matches				
			*n* train	*n* test	Field fluorescence (RFU)				Laboratory chl-a (µg L^−1^)		*n*
					Mean	SD	Mean	SD	Mean	SD	
8/31/2021	9/2/2021	− 2	212	74	1.41	0.42	1.26	0.11	8.32	1.15	3
9/9/2021	9/12/2021	− 3	174	60	0.81	0.09	0.52	0.28	1.57	1.07	3
10/14/2021	10/14/2021	0	142	50	0.89	0.10	0.78	0.18	1.69	1.06	6
8/3/2022	8/3/2022	0	725	244	0.95	0.75	0.88	0.60	6.92	3.62	9
*8/25/2022*	–						1.10	1.68	7.30	5.62	10
9/27/2022	9/27/2022	0	371	126	0.23	0.14	0.15	0.12	3.22	1.75	7
10/6/2022	10/7/2022	− 1	793	266	0.36	0.15	0.36	0.19	1.70	0.87	10
7/19/2023	7/19/2023	0	234	80	1.15	0.25	1.26	0.35	3.46	2.76	6
7/26/2023	7/26/2023	0	803	270	1.32	0.41	1.39	0.40	2.98	2.17	10
9/14/2023	9/14/2023	0	785	263	1.08	0.44	0.73	0.19	2.90	1.30	9
9/19/2023	9/19/2023	0	605	204	0.67	0.17	0.59	0.12	2.48	1.37	10
10/12/2023	10/12/2023	0	1058	356	0.78	0.14	0.79	0.15	2.12	0.94	9
All	–		7895		0.85	0.50	0.81	0.69	3.64	3.24	92

#### Satellite product evaluation

**Fig. 4:**
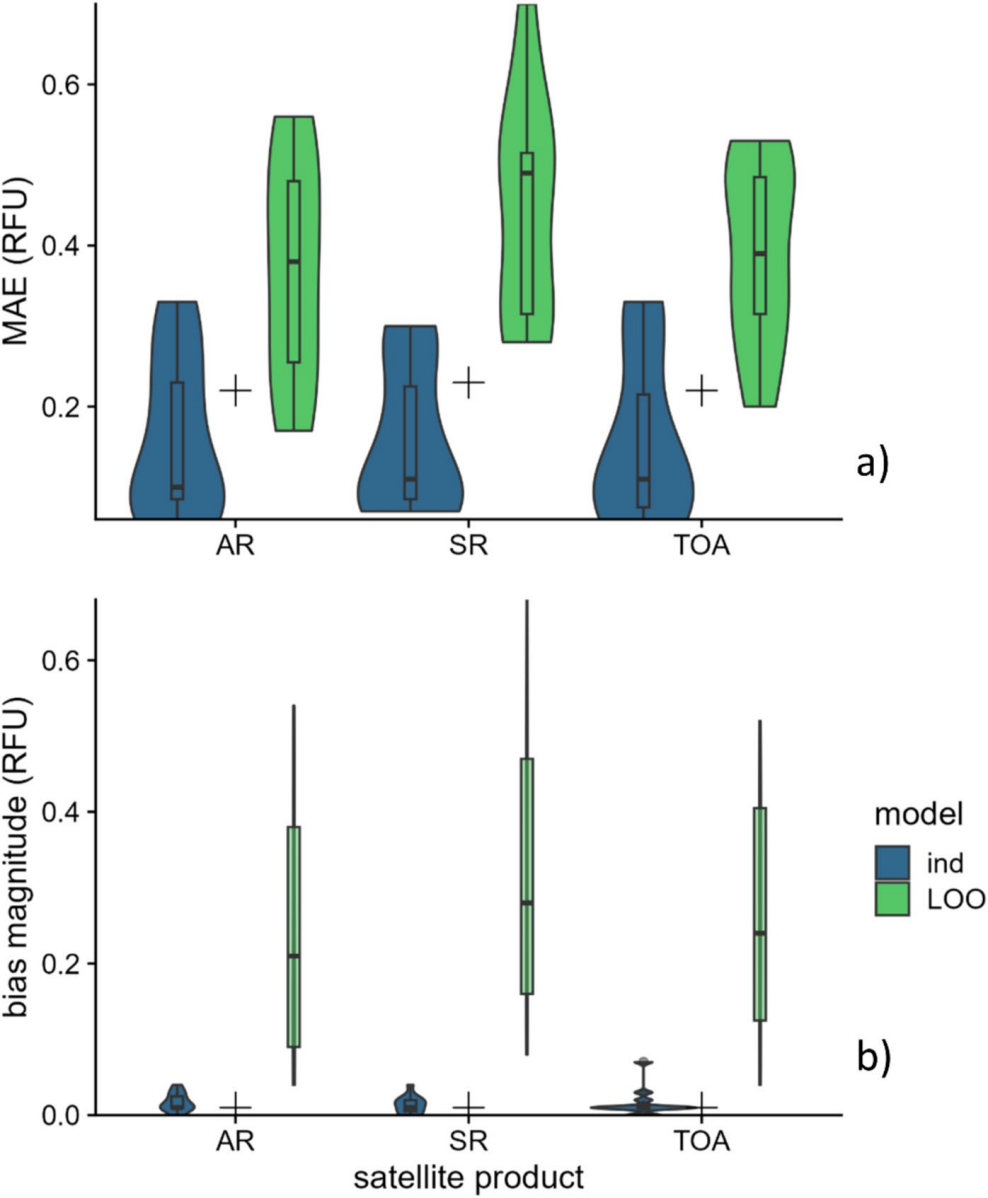
Distributions of **a** mean absolute error (MAE) and **b** bias magnitude, both in relative fluorescence units (RFU), for the individual (ind) and leave-one-out (LOO) models across the 11 sampling days for each of the three satellite products. “ + ” symbols represent values for the pooled model for all days for each product. Boxes (overlaid on violin density plots) indicate interquartile ranges (IQR), whiskers indicate minima and maxima up to 1.5 × IQR outside of IQR, and points indicate values beyond 1.5 × IQR outside of IQR

Generally, we did not observe substantial differences in performance among the three satellite products (Table 3, Fig. 4, Appendix 1: Table 4, Fig. 9). For individual day models, mean and median MAE and bias magnitude were nearly identical among the three products, and bias magnitude mean rankings varied little. TOA tended to be ranked more favorably based on MAE by a small margin, with a mean rank of 1.55 versus 1.91 for both other products. For the pooled model for all days, MAE and bias were nearly identical among the three products.

**Table 3:** Summary of model performance for each model and product aggregated across days. For the pooled model for all days, there was only one model for each product, so statistics shown are raw values, not aggregates. Absolute values were used prior to mean and median calculations to express central tendencies of bias magnitude. Satellite products were ranked within each model type using mean absolute error (MAE) and bias magnitude; mean ranks are shown. Statistics of best performing product within each model type are bolded

Model	Product	Mean MAE	Median MAE	Mean |bias|	Median |bias|	Mean MAE rank	Mean bias rank
ind	AR	0.16	**0.10**	0.02	0.01	1.91	1.73
	SR	0.16	0.11	**0.01**	0.01	1.91	**1.64**
	TOA	0.16	0.11	0.02	0.01	**1.55**	**1.64**
LOO	AR	**0.37**	**0.38**	**0.24**	**0.21**	**1.45**	**1.36**
	SR	0.45	0.49	0.33	0.28	2.36	2.55
	TOA	0.39	0.39	0.26	0.24	2.09	1.73
All days	AR	**0.22**		0.01		*NA*	
	SR	0.23		0.01			
	TOA	**0.22**		0.01

We observed small differences in performance among the three products for LOO models. Specifically, SR tended to exhibit slightly higher MAE and bias than AR and TOA. AR and TOA performed similarly, though AR had the best performance in all metrics by small margins (< 0.04 RFU). Though differences in error among products tended to be small, there were two exceptions: the August and September 2021 results yielded notably lower error for TOA and AR, respectively, and high error for SR.

For concision in reporting some subsequent analyses (model performance regression plotting, variable importance, mapping of modeled chl-a fluorescence), we focus on one satellite product, choosing AR given its slightly lower error rates for the LOO models (in the absence of notable differences for individual day models and the pooled model for all days).

#### Model type evaluation

**Fig. 5:**
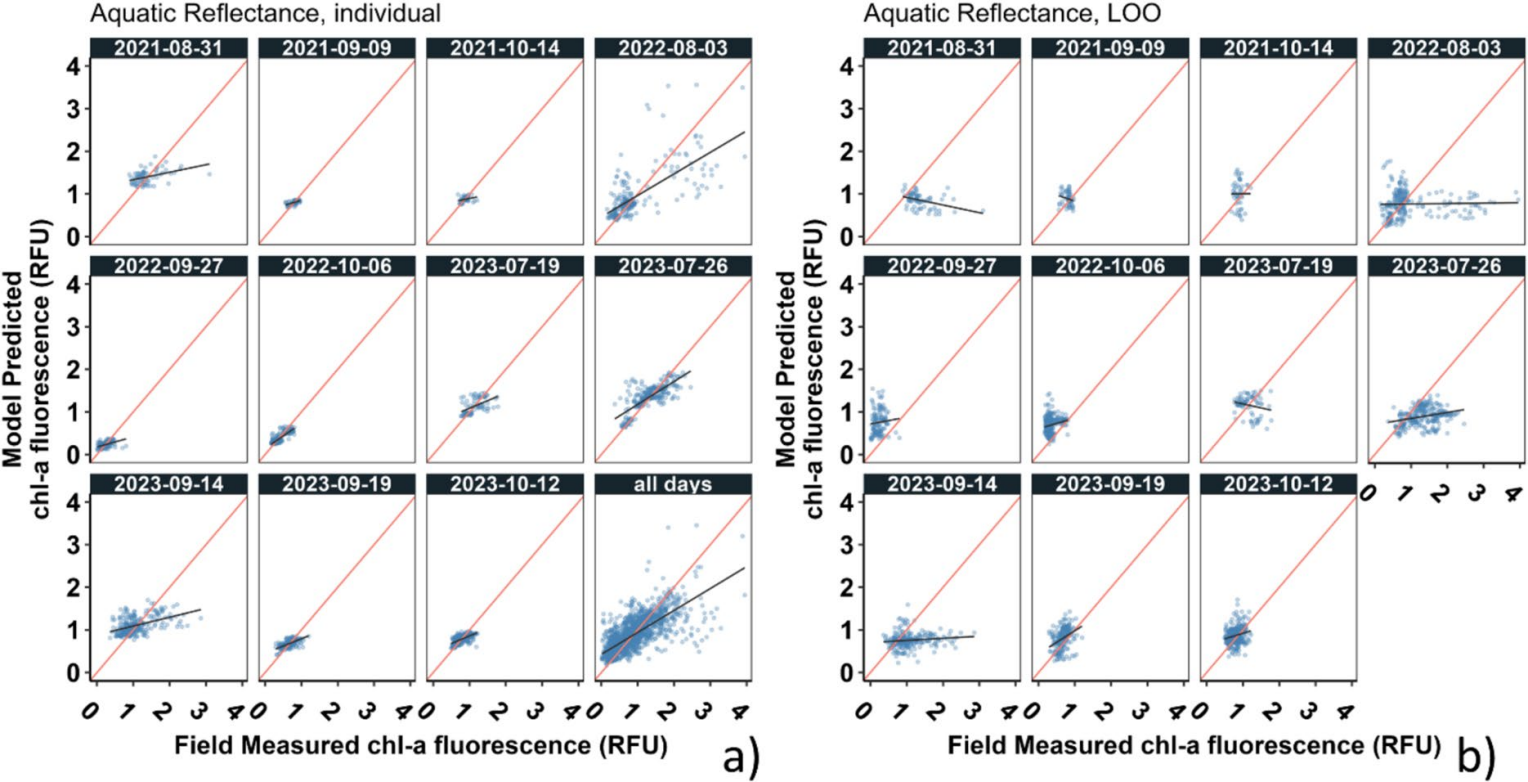
Scatterplots showing test performance of individual day models and the pooled model for all days (**a**), and leave-one-out (LOO) models on each day (**b**), for aquatic reflectance (AR). Individual day models were trained using 75% of data from a given day and tested on the remaining 25%; LOO models were trained using data from all other days and tested using only the same 25% of that day’s data; this 25% was the same set used to test that day’s individual model. 1:1 lines are shown in red

Without exception, individual day models outperformed LOO models. This was true in terms of both MAE and bias magnitude for all satellite products on all days (Table 3, Figs. 4 and 5, Appendix 1: Table 4, Figs. 9 and 10). The discrepancy was particularly notable in bias magnitude (Fig. 4b). Both types of models tended to overestimate low values and underestimate high values, as indicated by a general tendency for regression fit lines to be shallower than 1:1 lines in performance evaluation scatter plots (Fig. 5, Appendix 1: Fig. 10); however, LOO models were generally less responsive than daily models across the range of field-measured chl-a fluorescence, further indicating poorer fit. The pooled model for all days performed better than LOO in most cases but generally worse than individual day models (Table 3, Fig. 4). Eight of the 11 individual day models exhibited lower MAE than the pooled model for all days across all three satellite products (Appendix 1: Table 4).

**Fig. 6:**
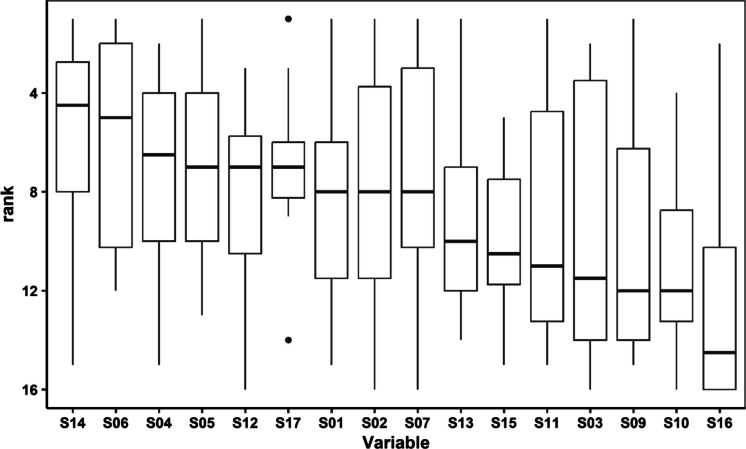
Summaries of variable importance ranks for each of the 17 spectral indices that were used as predictor variables, ordered by median rank, across all individual daily random forest models for AR. Boxes indicate interquartile ranges (IQR), whiskers indicate minima and maxima up to 1.5 × IQR outside of IQR, and points indicate values beyond 1.5 × IQR outside of IQR. Refer to Table 1 for spectral index algorithm specifications

**Fig. 7:**
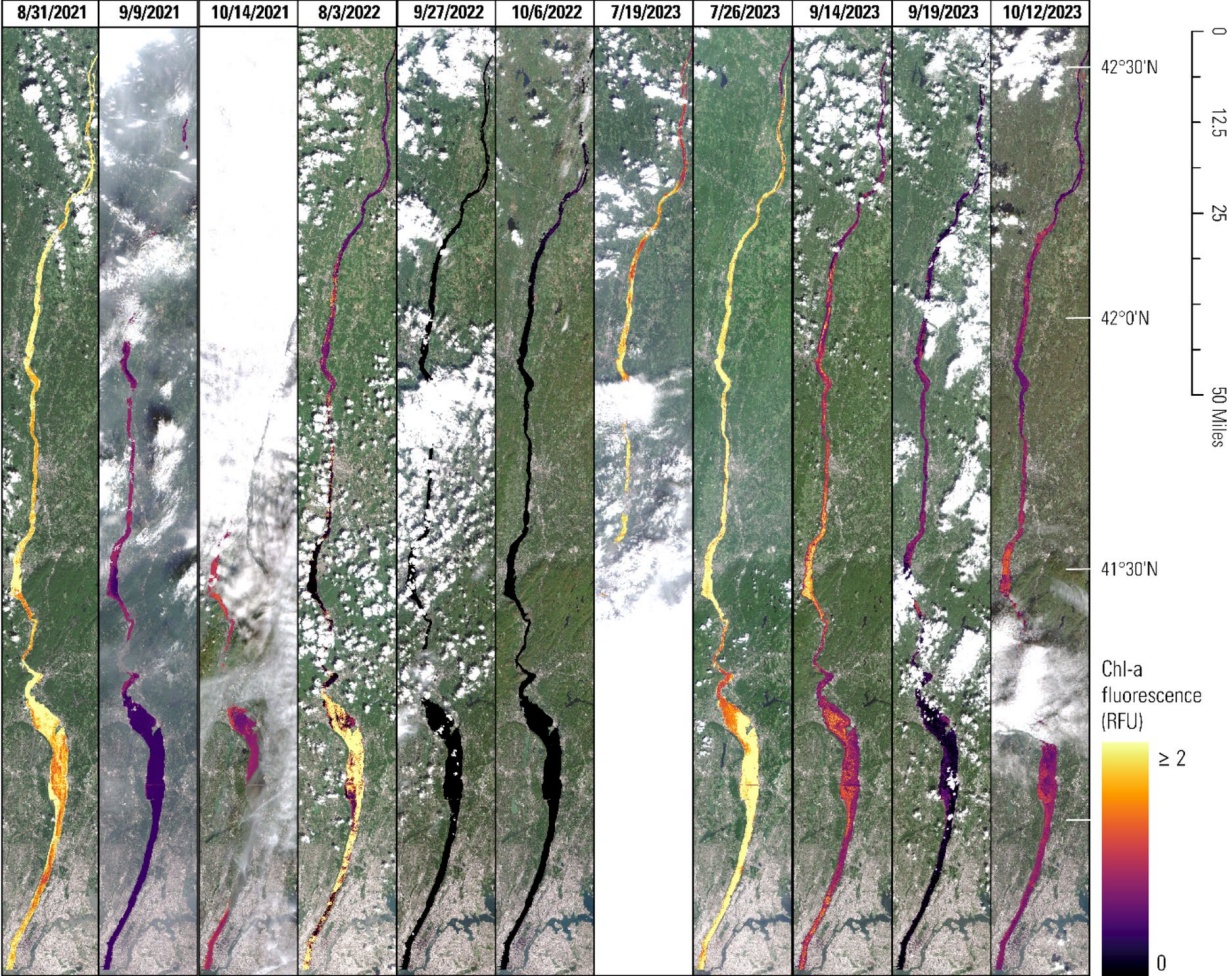
Modeled chlorophyll-*a* (chl-a) fluorescence (in RFU) during survey dates throughout the lower Hudson River, New York, generated by applying AR individual day models to satellite imagery obtained via the Copernicus Application Programming Interface (ESA, 2022)

#### Variable importance

Variable importance was calculated for each individual day model. For each day, using these importance values, each spectral index predictor was assigned a rank, 1 being most important and 17 being least important. We performed this analysis for each remote sensing product. Ranks for each predictor across all individual day models are summarized for AR in Fig. 6 and for SR and TOA in Fig. 11a and b, respectively, in Appendix 1. For AR, S14 and S06 were ranked highest across individual day models, with nearly the same median rank (5), followed by a substantial drop in median importance for subsequent variables (> 7). For SR, the top ranked indices were S14, S06, S02, and S07 after which median importance dropped (> 7, compared to < 5). For TOA, S02 had the highest median rank, followed by S06, S07, and S14. Median ranks for all other predictors were substantially lower (median rank ≥ 8, compared to ≤ 4).

### Mapping

Once chl-a fluorescence models are developed, they can be applied to spectral data to estimate chl-a fluorescence values throughout the lower Hudson River. We show examples of modeled chl-a fluorescence using AR across all non-cloudy river pixels for each of the 11 survey dates (Fig. 7). Several dates were affected by cloud cover in certain regions of the study area, which was masked from training, testing, and demonstrating the model. Some potential anomalies are visible along shorelines—for example, lower values along the shoreline of the middle reaches in the image from 7/26/2023. These may stem from presence of emergent aquatic vegetation, bottom reflectance, exposed tidal flats, or other shoreline phenomena not removed by the Sentinel-2 scene classification layer. These anomalies did not affect observations used for model training and testing because all data were collected away from shore in the shipping channel, but they demonstrate a data quality consideration that may be reduced by removing pixels near shorelines.

Spatial and temporal variability are readily apparent across the time series, though temporal variability appears greater than spatial variability in the lower Hudson River during the survey dates. The mean of standard deviations across individual dates was 0.23 RFU (mean range = 1.59 RFU), whereas the pooled standard deviation of all values across space and time was 0.54 RFU (pooled range = 4.61), indicating that temporal variability across the 3-year time series tended to be greater than the spatial variability across the lower Hudson River observed on any given day. Median values varied by roughly half an order of magnitude across dates. The field-measured RFU values supported this pattern, showing mean of standard deviations across dates of 0.28 RFU and pooled standard deviation of 0.50 RFU.

The image from 8/3/2022 showed the most spatial variability, with a standard deviation of 0.74 RFU—far greater than the next most variable image (7/26/2023), which had a standard deviation of 0.36 RFU. The two dates with the highest median modeled chl-a fluorescence throughout the river were 7/26/2023 and 8/31/2021 (1.45 and 1.38 RFU, respectively), while 9/27/2022 had the lowest median (0.23 RFU).

## Discussion

We developed and applied an approach to monitor chl-a fluorescence in the lower Hudson River using remotely sensed data. We sampled 12 dates across 3 years during summer and fall months; the range of chl-a values represented in our study is within the range typically observed in the Hudson River (Graham et al., 2022; Myers et al., 2024). We offer insights into nuances of modeling chl-a using remotely sensed data, building on previous work that developed and demonstrated use of a suite of spectral indices to predict chl-a occurrence, e.g., Saberioon et al. (2020). Our approach is unique in that we used chl-a fluorescence measured in RFU as the response variable, rather than chl-a concentration measured in µg L^−1^ or mg m^−3^, which is often the response variable targeted in chl-a modeling efforts using satellite algorithms (Gupana et al., 2021; Pahlevan et al., 2020; Salls et al., 2024; Seegers et al., 2021).

Targeting chl-a fluorescence offers the ability to leverage vast amounts of data collected in routine monitoring efforts. In situ field measurement of chl-a via fluorescence has emerged as a common practice; extensive data have been collected using this approach, including from sensors deployed at stationary monitoring sites that can offer temporally dense datasets (Stackpoole et al., 2024). Often, these data are accompanied by few or no comparative laboratory analyses (Foster et al., 2022; Suggett et al., 2010). The process of sample collection and laboratory extraction to quantify chl-a concentration tends to be costly and time-consuming, prohibiting rapid, cost-efficient, and most importantly spatiotemporally rich data collection. By using field-measured fluorescence, we were able to obtain thousands of observations for model training and testing. Importantly, we opted not to use the µg L^−1^ chl-a concentration units offered by the YSI Total Algae Sensor because these have been shown to be highly dependent on a variety of factors and are often unreliable (Foster et al., 2017; Liu & Georgakakos, 2021; Roesler et al., 2017), potentially providing misleading results if interpreted as absolute chl-a values. By choosing fluorescence as the response variable in our satellite models, we target the innate parameter reported by the field instrument—an optical response potentially more aligned with the signal leveraged from satellite sensors. We sought to develop and employ a method to explore relative spatiotemporal variability throughout the study system, which can be assessed using chl-a fluorescence without conversion to concentration in µg L^−1^.

The risk of using chl-a fluorescence as a modeled response is the same as for any use of chl-a fluorescence: the relation between fluorescence and actual chl-a concentration is often highly variable. This relation is variable for several reasons, including the matrix effects of other materials that interact with light, such as dissolved organic matter and suspended particulate matter, non-photochemical quenching, and algal community composition, light history, and health (Foster et al., 2022). Modeled chl-a fluorescence response could be converted to µg L^−1^ values; however, in our dataset, the relative strength of the relation between field-measured chl-a fluorescence and laboratory-measured chl-a values was relatively poor (*r*^2^ = 0.25; Fig. 3a) and conversion would introduce a substantial amount of error. The strength of the field-measured fluorescence and laboratory-measured chl-a relation varies among other studies. For example, Chaffin et al. (2021) reported *r* = 0.54 (equivalent to *r*^2^ = 0.29) for two sampling dates across over a hundred sites in Lake Erie. Prestigiacomo et al. (2022) investigated nearly 2000 paired observations for lakes in the State of New York, reporting a weak relation (*r*^2^ = 0.34) that became much stronger (*r*^2^ = 0.85) after removal of 24 outliers. Liu and Georgakakos (2021) reported *r*^2^ = 0.72 over 3 years across hundreds of lakes in the southeastern U.S. using other in situ water quality parameters to correct for biases in chl-a fluorescence; after evaluating data by year, relations were even stronger (up to *r*^2^ = 0.80). Similarly, when we evaluated our data by individual day, relations tended to be much stronger (up to *r*^2^ = 0.91). In cases where the ultimate target is chl-a concentration, this source of uncertainty across time adds to the uncertainty present when applying models to unseen days. Regardless of which response variable we were to select in this study, satellite sensors are retrieving a series of optical signals—though related, spectral response is a fundamentally different parameter than chl-a concentration or fluorescence, which are also different from each other. Ultimately, satellite measurements are based on light and therefore may have greater potential to relate to fluorescence than chl-a concentration; assessing the extent to which this is true was outside the scope of this study, but may be relevant for future exploration. Remote sensing applications have been applied in rivers, lakes, and estuaries around the world (Matthews, 2011; Yang et al., 2022). However, contextualizing the performance of our models relative to other studies is difficult. Few, if any, satellite studies target chl-a fluorescence in RFU as a response variable, instead focusing on chl-a concentration values in µg L^−1^, the variable routinely targeted in management and regulatory thresholds. Since our chosen error metrics MAE and bias are reported in the native units of the response variable (RFU), direct performance comparisons with studies focusing on chl-a concentrations are difficult.

We investigated three types of models in this study: (1) individual models trained for specific days, (2) LOO models, each trained on all days except one reserved for testing, and (3) a pooled model trained using all days, with 25% of data reserved for testing. Commonly, studies validating satellite algorithms use the latter approach, wherein all data from all sites and dates are pooled together for analysis (Harvey et al., 2015; Johansen et al., 2024; Saberioon et al., 2020; Salls et al., 2024; Seegers et al., 2021). However, our results indicate that this approach may overestimate the model performance that can be expected when applying a given model to unseen data.

The LOO model type investigated here demonstrates how a pooled model is likely to perform on any given single day not seen in the training set. Though both LOO and pooled model types were trained on nearly the same data (less one of 11 days), the pooled model was tested on all days in aggregate. Thus, the distributions of RFU values in training and testing sets were similar in the pooled model—especially given stratification of chl-a values prior to splitting—while LOO models were much more likely to encounter different distributions in training and testing data. In practice, when a model is trained on available data with the intent of applying the model to future (i.e., unseen) days, there is the same risk: the conditions on a future day may be different from those represented in the testing data, leading to model error. In systems that are temporally homogeneous, the difference between pooled and LOO models is likely to be small because any given day is likely to share similar conditions as those in the training data; in the highly temporally variable Hudson River, though, the difference is notable. We thus conclude that, in our system, the LOO model assessment provides a more conservative assessment of model performance than the pooled model. However, given that our study investigated only a single system, further study would be needed to determine whether the same statement can be made in larger studies spanning multiple systems.

We acknowledge that our approach to test the LOO models differs from the approach that would be employed when applying such a model in practice: we used only 25% of each test day, testing against the same set of observations used to test individual daily models and the pooled model, thus enabling comparison of error metrics between model types. Our purpose in presenting both pooled and LOO modeling results was to demonstrate two different methods of building models on multiple days of training data that could be applied to estimate chl-a fluorescence for future days. Given that we observed notably different levels of error between the two methods, our results demonstrate the importance of carefully considering an approach that suits project needs, and of fully understanding modeling methods when making comparisons across studies. Additionally, the difference in performance between the two approaches suggests that these models suffer from insufficient inclusion of the range of system characteristics that may be present on a given day. Inclusion of more data—specifically, from more points in time—may help model performance. Parity of LOO and pooled model performance would suggest that system variability was more adequately captured in training data, making models more likely to succeed.

**Fig. 8:**
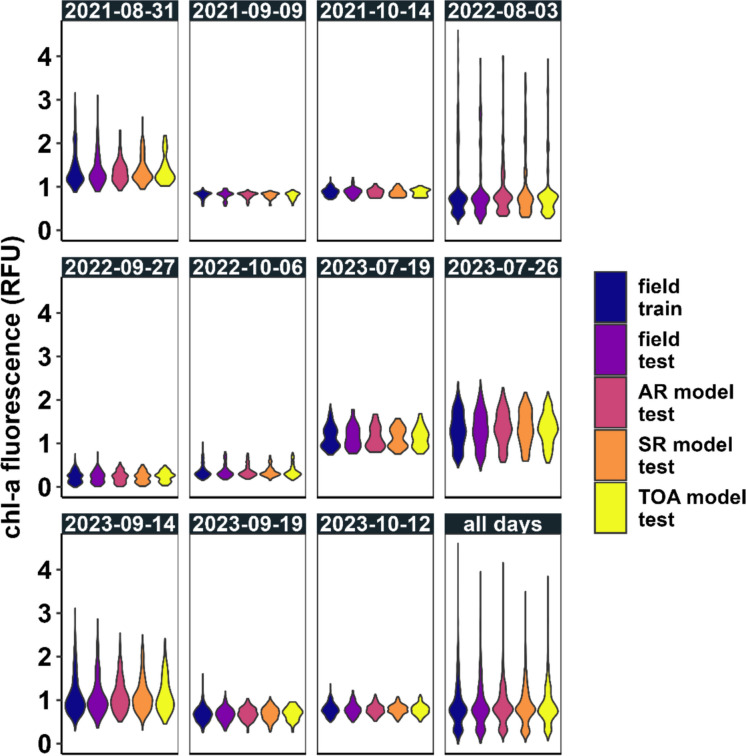
Distributions of field-measured chlorophyll-*a* (chl-a) relative fluorescence values (RFU) used for training and testing, and of satellite modeled chl-a fluorescence for each satellite product—aquatic reflectance (AR), surface reflectance (SR), and top of atmosphere (TOA)—for each individual day model and for the pooled model for all days

Individual day models performed better than LOO models in all cases and better than the pooled model for all days in most cases. This approach is not common practice, though training a new model for each day of satellite imagery is extraneous and usually impossible when field data are not collected on a regular basis. However, if the objective of remote sensing monitoring is to extrapolate chl-a values between sites for dates with existing field data, individual day models appear to generate more accurate estimates, on average, than pooling all data into a single model. Individual day models appear to benefit by adapting to atmospheric conditions and matrix interference effects that vary between dates. This finding also suggests that, in systems with spatially dense networks of deployed continuous sensors, such as the HRECOS network on the lower Hudson River (*Hudson River Environmental Conditions Observing System*, 2023), it may be possible to train daily models that perform better than a single model for all days to extrapolate chl-a estimates across the system beyond monitoring sites. For example, chl-a estimates throughout the lower Hudson River (e.g., Fig. 7) may be generated for each satellite overpass using unique daily models. However, such an approach would likely only be practical in systems where continuous sensors are already deployed and not in cases where additional field collection efforts would be required. Less frequent field data collection is a major impetus for employing satellite remote sensing into monitoring programs; collecting field data for every date of satellite overpass is infeasible. Also, in cases of relatively low spatial variability, such as presented in Fig. 7, efforts to make extrapolations solely in space (and not in time) add limited value. Still, our work suggests that exploring the use of existing continuous monitoring networks to train daily models could add value to monitoring efforts. The poor performance of the LOO models relative to the daily models suggests that temporal variability is an important source of uncertainty in the lower Hudson River. Our study system exhibited more variability in time than in space, as indicated by both field measurements and modeled satellite values (Table 2, Fig. 7); thus, the conditions on a given day may vary substantially from “normal” conditions established by the training set. In our training and testing data, the respective distributions of chl-a fluorescence values were similar within a given day but varied greatly across days (Appendix 1: Fig. 8), indicating that a given LOO model could have different distributions between training and testing data, potentially impairing performance on the test day. This risk is unavoidable when applying a model to a day unseen in training, which is a reality of most remote sensing monitoring applications whose impact can be reduced by collecting data from additional days in attempt to capture the full range of variability in the system across time. Our finding that removal of even 1 day of training data can substantially impact performance underscores importance of selecting training data that fully represents system variability during the time horizon desired for making estimates—whether it is a single day, or across many years. It is also likely that the models were confounded by other factors that vary across time. Most notably, these include (1) atmospheric conditions, including thin clouds not removed by the cloud mask (Coluzzi et al., 2018) which appear to be present in some images shown in Fig. 7; (2) factors that influence chlorophyll fluorescence response, such as temperature, light environment, and algal community composition and health (Foster et al., 2022); and (3) other optically active water constituents (dissolved organic matter, suspended particulate matter). We measured fDOM and turbidity via sonde during boat surveys; while we did not observe any relation between daily model error and daily means of either of these parameters (results not shown), these and other water quality parameters may create variability in the relation between optical signals present in satellite data and fluorescence measured in the field, explaining variations in model performance across dates. Although not described in this study, the dataset we used also contained concurrent phytoplankton taxonomic data. Available data (Welk et al., 2025) suggested a positive relation between model error and prevalence of small taxa (< 9 µm) and detritus in samples. The spectral response captured in satellite data and used in this study did not account for differences in taxa, but it is well established that differences in phytoplankton community can create different fluorescent responses. This disconnect could explain some variability we observed in relations between days and may explain some model error in general. While more in-depth analysis on this topic was outside the scope of this study, these data indicate that further investigation into the relation between phytoplankton taxa and model performance is warranted. Together, the same interferences in the chl-a-to-fluorescence relation likely also interfere in satellite retrieval of fluorescence, further limiting the ability of these models to be used for chl-a concentration; utility lies in assessing relative changes in fluorescence, which may serve as a proxy for phytoplankton abundance.

**Fig. 9:**
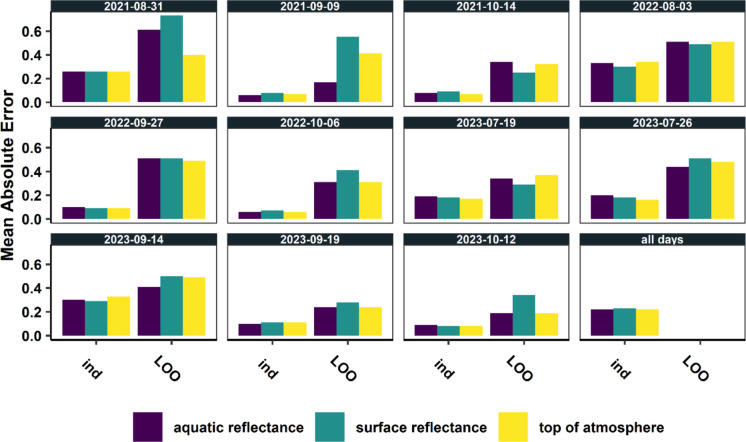
Mean absolute error (MAE, in relative fluorescence units [RFU]) by survey date for each of the three satellite products, for individual date (ind) and leave-one-out (LOO) pooled models; the final plot shows MAE for the pooled model for all dates

**Fig. 10:**
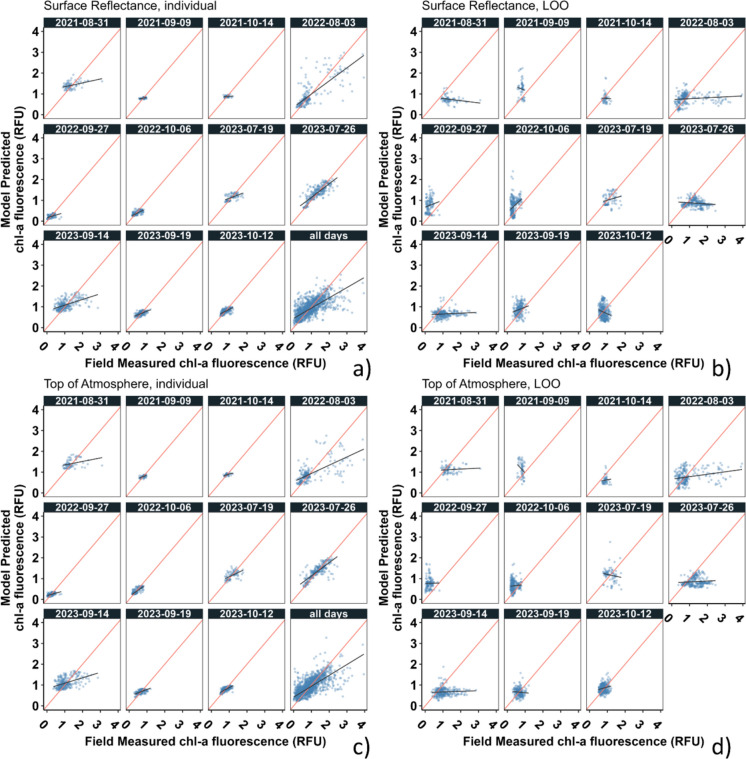
Scatterplots showing test performance for surface reflectance (SR) of individual day models on each day and the pooled model for all days (**a**) and leave-one-out (LOO) models (**b**) on each day, and same for top of atmosphere (TOA) (**c** and **d**, respectively). 1:1 lines are shown in red

**Fig. 11:**
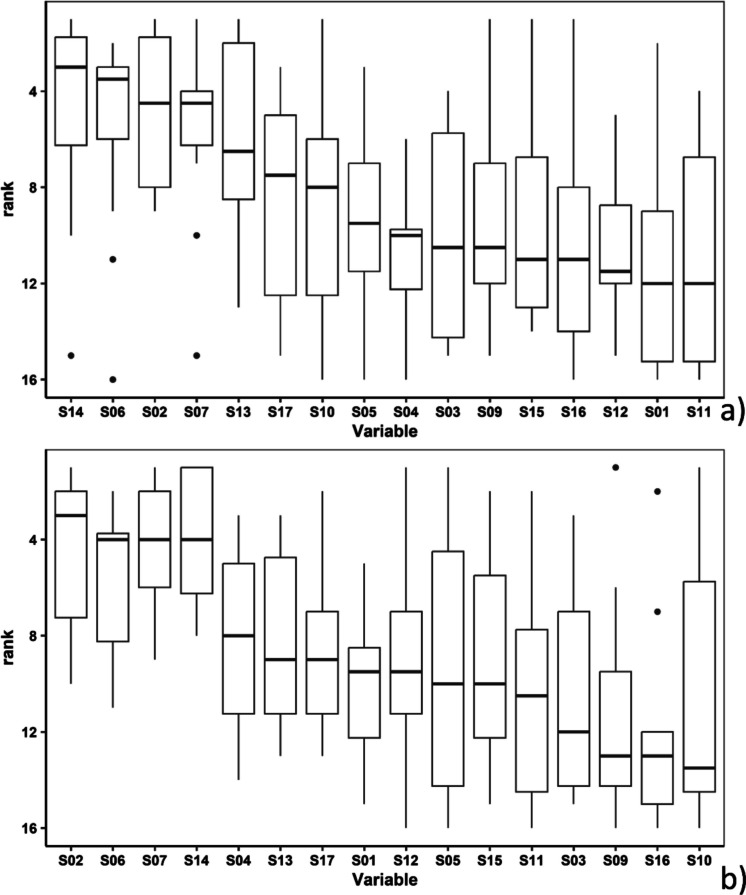
Summaries of variable importance ranks for each of the 17 spectral indices that were used as predictor variables, ordered by median rank, across all individual daily random forest models for **a** SR and **b** TOA. Boxes indicate interquartile ranges (IQR), whiskers indicate minima and maxima up to 1.5 × IQR outside of IQR, and points indicate values beyond 1.5 × IQR outside of IQR. Refer to Table 1 for spectral index algorithm specifications

Choice of satellite product did not strongly influence model performance, particularly for individual day models and the pooled model for all days. We observed slight differences for LOO models, which may be at least partially attributable to the higher error magnitudes in these models that may have exacerbated minor performance differences, though these differences were still relatively small. SR exhibited relatively lower error on days with the heaviest cloud cover (10/14/21 and 7/19/23) and error comparable to other satellite products for some days with moderate clouds (8/3/22 and 9/27/22; Figs. 7 and 9); further study is required to determine whether this pattern occurs systematically in other datasets. When daily models were used, we observed almost no differences in model performance between products; the variability in atmospheric and water column conditions appears to be well captured in the training for a given day, and the choice of satellite product appears inconsequential. When training models on data from multiple days to make estimates on novel days, as for the LOO models, differences may result from choice of atmospheric correction. In our study, AR showed slightly lower error than SR and TOA in this scenario, though choice of satellite product did not influence performance dramatically in our dataset. Note that we refrain from making a specific recommendation on choice of satellite product.

Variable importance ranks for individual day models provide insight into which algorithms—and based on these, which wavebands—tended to explain the most variance in chl-a fluorescence. For all three satellite products, our aggregation of importance ranks across the 11 daily models consistently showed S14 and S06 to be among the most important variables based on median rank. S02 and S07 were both in the top four most important variables for TOA and SR. All four of these algorithms use MSI bands 2 and 3, and three of the four also use band 4, corresponding to blue, green, and red wavelengths, respectively. Generally, the shorter wavelengths are thought to be more influenced by colored dissolved organic matter (Gholizadeh et al., 2016), as uncertainties introduced by atmospheric constituents, and when utilized, atmospheric correction (Pahlevan et al., 2021). As a result, many remote sensing efforts use longer red, red-edge, and near-infrared wavelengths in algorithms such as the MCI and NDCI. In our study, the temporally varying influences of these uncertainties may have been minimized in the individual day models. Some algorithms leveraging longer wavelength bands appear to exhibit minimum detection limits around 5 or 10 µg L^−1^ (Binding et al., 2013; Gons et al., 2008; Salls et al., 2024). Given that most laboratory-measured chl-a values in our study were near or below this threshold, algorithms leveraging red and red-edge bands may become more important in systems with higher chl-a values shown to be more successfully retrieved using these wavelengths.

### Future work

Future work may seek to test the use of stationary near-surface deployed sondes, as opposed to boat survey transects, to train satellite models in the lower Hudson River and other locations with sensor networks. Our results indicate that variability between days is a greater barrier to model performance than data scarcity, suggesting that temporally specific models trained with fewer data may be successful in areas that already have networks of deployed continuous sensors. Further work could help understand and account for the effects of interfering water constituents—such as dissolved organic matter, suspended particulate matter, and their changes across time. Our investigation revealed no relation between magnitudes of error and these parameters, but more in-depth modeling efforts may reveal different patterns. Inclusion of parameters like colored dissolved organic matter and turbidity—whether derived from satellite measurements or collected via continuous sensors—as additional predictors in models using satellite data to estimate chl-a may help account for variability in relations between optical signals and chl-a, potentially improving model performance. Better understanding the impact of varying phytoplankton community characteristics on satellite retrievals may also enable construction of more effective models. Finally, our study did not employ adjacency effect correction; with recent advances in this field, future work to test new correction algorithms may facilitate their adoption.

### Limitations

In this study, we developed models to predict chl-a fluorescence in RFU, not chl-a concentration. This approach enables comparisons across space and time while leveraging spatiotemporally rich field fluorometry data but has limited utility for regulatory applications or classification of trophic state. The methodology we present may be replicated in other study areas or expanded across broader regions but was developed based on data from the lower Hudson River and has only been tested there. Most dates in our study only had partial data coverage by field data (note some limited sample sizes in Fig. 3b), satellite imagery, or both—some field surveys spanned only part of the study area, and clouds obscured some areas of several images and may have interfered with accurate satellite retrievals (e.g., Fig. 7). Chl-a concentration and RFU ranges in our datasets were limited and low, representing conditions in the lower Hudson River during data collection; this limits the reliability of our models to estimate RFU for higher values. Building similar models in systems with greater variability may yield stronger performance. We were unable to assess the impact of latency between field measurement and satellite image collection times given the limited scope of our dataset because only three images were collected outside of the same day field measurements were taken; however, we did not observe appreciably higher error on those days. Still, given the relatively high temporal variability observed in the study system, changes in river conditions between satellite and field collections could be a factor contributing to error. We assessed three satellite products and reported performance of each as a response variable in models; however, this study is not intended to serve as a comprehensive assessment to inform choice of satellite product, but rather, to demonstrate that this choice did not appear to affect model performance substantially in our case. Likewise, our assessment of variable importance indicated which algorithms used as predictor variables explained the most variance in model response but was not intended to serve as an algorithm comparison.

## Conclusions

This study developed and tested a series of satellite models to estimate chl-a fluorescence in the lower Hudson River. We compared three modeling approaches, finding that (1) pooling data across dates and sites and splitting it into training and testing sets—a common approach in validation of satellite algorithms—may overestimate model performance in our study system, and (2) individually trained daily models are likely to yield the best performance based on MAE in cases when collected data are sufficiently temporally dense. We compared three satellite products, finding that performance of AR was marginally better than TOA, and that both tended to perform better than SR. Ultimately, however, the choice of product did not substantially impact performance. Finally, we found that the relation between field-measured chl-a fluorescence and laboratory-measured chl-a was poor overall, but that splitting the data into individual days resulted in substantially stronger relations. Our results emphasize the importance of accounting for daily variability when scaling between point, transect, and surface estimates of river chl-a across observation methods.

## Data Availability

All data used in analyses described in this study are available in (Welk et al., 2025).

## Appendix 1. Ancillary information on model performance

Data sources for Appendix materials. Field fluorescence data are described in Welk et al. (2025). Satellite data were obtained via the Copernicus Application Programming Interface (ESA, 2022).

**Table 4:** Mean absolute error (MAE) and bias for individual models (ind), leave-one-out models (LOO), and the pooled model for all days across the three satellite products: aquatic reflectance (AR), surface reflectance (SR), and top-of-atmosphere (TOA). Units are in relative fluorescence units (RFU)

Date	ind						LOO					
	AR		SR		TOA		AR		SR		TOA	
	MAE	Bias	MAE	Bias	MAE	Bias	MAE	Bias	MAE	Bias	MAE	Bias
8/31/21	0.26	− 0.02	0.27	0	0.26	0	0.56	− 0.54	0.7	− 0.68	0.32	− 0.23
9/9/21	0.06	0	0.07	− 0.01	0.07	− 0.01	0.17	0.07	0.59	0.51	0.48	0.38
10/14/21	0.08	− 0.01	0.09	− 0.02	0.07	− 0.01	0.33	0.13	0.28	− 0.14	0.35	− 0.32
8/3/22	0.33	− 0.03	0.3	0	0.33	− 0.07	0.54	− 0.21	0.51	− 0.23	0.52	− 0.2
9/27/22	0.1	− 0.01	0.09	− 0.01	0.1	0.01	0.5	0.49	0.51	0.49	0.53	0.52
10/6/22	0.06	− 0.01	0.07	− 0.02	0.06	0	0.28	0.23	0.38	0.28	0.31	0.24
7/19/23	0.19	− 0.02	0.18	− 0.02	0.17	− 0.01	0.38	0.08	0.3	− 0.08	0.39	0.05
7/26/23	0.2	0.04	0.18	0.04	0.16	0.01	0.46	− 0.43	0.52	− 0.45	0.49	− 0.45
9/14/23	0.3	0.03	0.29	0.02	0.32	0.03	0.41	− 0.33	0.49	− 0.45	0.47	− 0.43
9/19/23	0.1	0.01	0.11	0.01	0.11	0.02	0.23	0.1	0.29	0.18	0.24	− 0.05
10/12/23	0.09	− 0.01	0.08	0	0.08	− 0.01	0.2	0.04	0.33	− 0.09	0.2	0.04
All days	0.22	0.01	0.23	0.01	0.22	0.01						
